# Government Communication, Perceptions of COVID-19, and Vaccination Intention: A Multi-Group Comparison in China

**DOI:** 10.3389/fpsyg.2021.783374

**Published:** 2022-01-21

**Authors:** Linsen Su, Juana Du, Zhitao Du

**Affiliations:** ^1^School of Language and Communication, Beijing Jiaotong University, Beijing, China; ^2^School of Communication and culture, Royal Roads University, Victoria, BC, Canada; ^3^School of Journalism and Communication, University of Chinese Academy of Social Sciences (UCASS), Beijing, China

**Keywords:** health belief model, government communication, perceived severity, COVID-19, vaccination intention, perceived susceptibility, perceived benefits, perceived barriers

## Abstract

Government communication has been playing an important role in mass vaccination to conduct the largest vaccination campaign of the world for COVID-19 and to counter vaccine hesitancy. This study employs the health belief model to examine the association between government communication and the COVID-19 vaccination intention. A survey of Chinese adults (*N* = 557) was conducted in March 2021, and partial least squares structural equation modeling was employed to estimate the multi-construct relationships. The findings indicate that government communication has both direct positive association with vaccination intention and indirect association with vaccination intention through the mediation of perceived severity, benefits, and barriers. Multi-group comparisons suggest that individuals from private sectors are more easily mobilized to receive COVID-19 vaccination by government communication than those from public sectors. Similarly, the correlation between government communication and the vaccination intention of individuals with a good health status was stronger than that of those with a poor health status. The theoretical and practical implications of these findings are further discussed.

## Introduction

Vaccine popularization is the most effective method to reduce the spread of an infectious disease. Vaccines are estimated to prevent 2 to 3 million deaths per year, and an additional 1.5 million lives could be saved if vaccination coverage can be further optimized (Feemster, 2020). There is an increasing consensus within the scientific community that the most effective method of defeating the COVID-19 pandemic is the mass vaccination of individuals worldwide. The vaccination of COVID-19 represents the biggest vaccination campaign in history. According to data collected by Bloomberg (2021), as of November 6, 2021, more than 7.2 billion doses have been administered across 184 countries.

Governments have consistently played a critical role in shaping attitudes of individuals toward vaccination (Wang et al., 2018). Governments have leveraged their capability to mobilize individuals to follow preventative rules (mask-wearing, applying hygiene rules, and social and physical distancing) in the fight against COVID-19. In addition, governments are essential in providing detailed information to build the trust of individuals in the safety and efficacy of vaccines in the face of hesitancy, contradictions, or resistance through intensive communication of the benefits and safety of vaccination (OECD, 2021). To mobilize the public to join forces in the collective actions against COVID-19, governments can efficiently communicate through media. Recently, numerous established and newly emerging online platforms have been playing increasingly important roles in government communication with audiences, and there have been accumulated reports of accelerated digital transformation in public administration institutions during the COVID-19 pandemic (Gabryelczyk, 2020). Therefore, government communication, especially communication through emerging new media outlets, is becoming omnipresent, thus, shaping the perception of the pandemic, vaccines, and the subsequent vaccination intention of the public.

The health belief model (HBM) has been widely employed as a comprehensive framework for analyzing the willingness of people to get vaccinated. However, several gaps remain in the current body of research. First, HBM seldom considered external factors beyond health or disease perception. For example, the communication strategies of the government may receive little attention despite the increasing studies of the role of governments in health communication campaigns during global pandemics (Rousseau et al., 2015; Li Y. L. et al., 2018; Duan et al., 2020). Second, the nuanced mechanism of mediation or moderation behind this influence is poorly understood (Carpenter, 2010; Jones et al., 2015). Third, specific groups of individuals susceptible to COVID-19 infection have gained little attention with regard to vaccination. For example, individuals with low health status are vulnerable to pandemic infection as herd immunity requires vaccination of a substantial proportion of the population (OECD, 2021).

As the most populous country with one of the lowest COVID-19 infection rates in the world, China, an authoritarian country, serves as an ideal setting to test the role of the government communication in fighting against COVID-19. This study investigates both direct and indirect associations (through the mediation of health-related beliefs) between government communication and vaccination intention and compares the role of government communication between the different groups. This study contributes to the literature in the following ways. First, it integrates the use of government communication into the conventional HBM model, which bridges the divide between mass communication and public health. Second, this study distinguishes the external cues of government communication. It further differentiates the role of government communication among individuals across different groups (within vs. outside the public sectors; poor vs. good health status). Third, it further investigates the role of health-related beliefs as mediators in the relationship between government communication and vaccination intention.

## Literature and Hypotheses

The role of the government in COVID-19 vaccination has garnered intensive research interest as this historically unprecedented vaccination campaign largely relies on the government regulation (Duan et al., 2020; OECD, 2021). Information channels of the government are regarded as one of the most important external cues that influence individuals to get vaccinated. Individuals are more likely to receive the vaccine after obtaining complete information and when vaccine uptake is more common among the public (Mahmud et al., 2021). Official media as a tool of government communication was utilized to mobilize the people in COVID-19 prevention in China (Duan et al., 2020). A growing number of individuals gained health information online. Social media, in particular, facilitates the information diffusion and promotes audience engagement. Therefore, some anti-vaccination conspiratorial information or other misinformation is prevalent in websites (Blume, 2006; Kata, 2012), especially on social media (Guidry et al., 2015; Featherstone et al., 2019). In contrast, the government employs official media in anti-COVID-19 mobilization efforts and propaganda, enhancing public confidence in vaccine safety and efficiency by releasing up-to-date research progress on COVID-19 vaccines.

Government communication during the anti-COVID-19 campaign carries special significance in China, where its collectivist culture meets the patriotic hygienic movement. The campaign-style health risk governance of China is characterized by its establishment of top-down leading groups or headquarters and complex horizontal institutional arrangements, all of which greatly increased its efficiency in decision-making and coordination in combatting the pandemic (Cai et al., 2021). Chinese authorities are employing a combination of pressure, incentives, and education to motivate mass vaccination to conduct the largest vaccination campaign of the world for COVID-19 to achieve herd immunity (Hua, 2021). Red banners with rhyming slogans have been employed in government communication for decades in China, which are now strung across the country and shared on social media in anti-COVID-19 and pro-vaccination campaigns (Xu and Renken, 2020). These banners project COVID-19 as a threat to personal health or others and to the state (Xu and Renken, 2020). Governments extensively use slogans in altruistic discourses. For example, some slogans read: “It is conscienceless to infect your parents with COVID-19 by returning home bringing viruses,” “Those who don’t report their fever (symptoms) are class enemies hiding among the people,” and “You get vaccinated, I get vaccinated, and everyone gets vaccinated, for healthier families and the society.” These slogans have received extensive attention online, and their impacts have been amplified through heated discussions on social media.

People from a collectivist culture tend to seek consensus and avoid conflicts with others, and Chinese individuals are highly collectivist and tend to think as a group (Hofstede, 2001). The Chinese have the tradition of linking nationalism or patriotism with health campaigns as the building of a sanitary system in China has been labeled as a patriotic movement against colonialism or imperialism (Rogaski, 2004). The Patriotic Public Health Committee at all levels of administrative agencies still exists in China today, ever since the Patriotic Hygiene Movement in the 1950s when China and North Korea accused the US of initiating germ warfare (Yang, 2004; Hu, 2013). Hygienic campaigns in China have frequently been associated with an anti-imperial nationalism and anti-colonial pride (Peng, 2020). Therefore, a perceived risk threatening national security would lead to a great hazard of concern to ordinary people in China (Xie et al., 2003), while the benefits to the nation, society, family, and others can enhance the persuasiveness of health campaigns in a collective culture such as China (Jia and Luo, 2021). Therefore, Chinese individuals often have a clearly defined sense of nationalistic expression, and this growing nationalism has been highlighted due to the successful control of the pandemic by the country (Liu, 2020). The strong Chinese nationalism is positively associated with counter-COVID-19 behavioral intention as the Chinese government has made tremendous efforts in an anti-pandemic mobilization (Jia and Luo, 2021). Chinese netizens display a strong sense of pride in the achievements of China in counter-COVID-19 campaigns, and express hostile sentiments when comparing China with other countries in the cyberspace (Zhao, 2020). The overall level of satisfaction of Chinese people toward the performance of the government during the COVID-19 pandemic is high, but the level of satisfaction decreases when the government becomes more local and less centralized (Wu et al., 2021). The national identity of Chinese is a combination of the love for family and people who share the same culture. For example, the title of a popular speech praising anti-COVID-19 campaign refers to “No country, no home,” and another short anti-COVID-19 slogan says “Home is the smallest country, and the country is made up of millions of homes. Only when there is a country, can there be a home. In the face of disaster, our country is our strong backer.”

People are encouraged by the government to get COVID-19 vaccines as soon as possible. In this sense, vaccination is a patriotic act in abiding by the suggestion of the government of “prevention is better than cure.” Hu et al. (2021) discovered that the Chinese people express collectivism-oriented opinions on COVID-19 vaccination and have favorable attitudes toward the COVID-19 immunity certificate (passport).

Knowledge of the government-media relationship is the key to systematically understand the function of the media in China (Winfield and Peng, 2005), and “state-market” paradigm is a core framework to study media system in the contemporary China (Li and Tang, 2012). There are two types of media under the party-market media system: party line media (official media) and market-oriented media (unofficial media). The two can be differentiated according to personnel, resources, political authority, and functional tasks (Zhao, 2008). The party line media plays a vital role in the Chinese media system and operate under the strict supervision of the Party propaganda departments, with their main function being to serve as the mouthpiece of the party (Zhao, 2008). The government extensively employs official media in vaccination propaganda, which would substantially mobilize individuals in the campaign. Subsequently, we propose the following hypothesis:

H1: Government communication is positively associated with COVID-19 vaccination intention.

The central notion of an insider-outsider theory is that the insiders (individuals with secure employment) can receive preferable access to excludable goods compared with outsiders (individuals without secure employment) (Rueda, 2005; Chang and Kerr, 2017). In China, the society is organized by a dual system of state-market. To apply the insider-outsider theory here, those affiliated with the state-sponsored institutions are “insiders” and the rest as the “outsiders.” Government communication, as a way to combat the pandemic, is uniquely positioned under authoritarian ways of governing in China. Almost all “insiders” (e.g., civil servants) work in the core of the system, whereas “outsiders” have no direct working relationship with the government or the Communist party (Naughton, 2016). Public servants, civil servants, teachers, and hospital employees work in the core of the party-state system and typically have the closest relationship with the government.

Individuals in government sectors (public sectors) are easily manipulated and tracked, whereas those outside the public sectors are relatively and loosely controlled by the government. The individuals in the public sectors are the implementers, operators, and beneficiaries of government policies, including government communication about COVID-19. Government communication is mainly initiated by the public servants who are asked to take a lead in implementing the counter-COVID-19 regulations. Similarly, individuals working in other public sectors, especially Communist party members, are asked to act as role models. However, individuals outside the public sectors receive less direct control from the government, thus, they are less likely to be tracked or mobilized compared to the insiders. The lack of direct control by the government further makes the outsiders less likely to adhere to COVID-19 preventive measures (e.g., COVID-19 vaccination), which provides a challenge to a massive vaccination campaign. Similarly, public servants and army or police officers in Indonesia, another Asian country with a collective culture, tend to more consistently abide by precautionary measures against COVID-19, e.g., wearing masks and washing hands, than others (Laksono et al., 2020). Therefore, we propose the following hypothesis:

H2: The association between government communication and vaccination intention is stronger for individuals in the public sectors than for those in the private sectors.

Numerous personal and demographic factors have been examined in relation to vaccination intention. Those factors include gender, age, health status, etc. In particular, researchers explored the impact of health status on vaccination intention. Studies confirmed that poor health status was strongly associated with low health literacy and vaccination intention. For instance, Chang (2011) found that adolescents with poor self-reported health status had low health literacy, and they were less likely to engage in health-promoting behaviors, including vaccination. Similarly, Suka et al. (2015) confirmed the positive relationships between health information access, health behavior, and health status in Japanese people, and they found that the individuals with sufficient health information were more likely to engage in a health behavior and report a good health status. In addition, researchers found that the high health literacy was further associated with a high preventative behavior intention, such as vaccination (Berkman et al., 2011; Castro-Sánchez et al., 2016). In terms of their health status, healthy individuals have a higher level of health literacy than the unhealthy individuals, and their attitudes toward vaccines are more positive than those of unhealthy individuals. Thus, health status is a critical factor that influences the degree to which the public is involved in preventive behaviors during a pandemic (Li and Liu, 2020). Healthy individuals also have stronger immunity to COVID-19 infections than unhealthy individuals. It is also found that the individuals with a poor health status reported high level of mental health problems (e.g., fear, stress) due to COVID-19 pandemic (Inbar and Shinan-Altman, 2021), and an “inadequate” or “problematic” health literacy was strongly associated with confusion about COVID-19 information (Okan et al., 2020). Individuals with a good health status are more health-conscious and tend to read more news on disease through government communication. Subsequently, they are more likely to be exposed to government communication than unhealthy respondents. As they tend to pay more attention to government communication related to COVID-19, they are also more immune to rumors and misinformation about COVID-19. Those results have practical implications on vaccine prioritization, and more understanding needs to be developed on the association between government communication and vaccination intention of groups of different health status. Based on the above discussions, we propose the following hypothesis:

H3: The association between government communication and vaccination intention is stronger for individuals with a good health status than for those with a poor health status.

The core components of the readiness to act of HBM advocate people is comprised of perceived susceptibility, perceived severity, perceived benefits, and perceived barriers (Garcia and Mann, 2003; Deshpande et al., 2009). Many studies examined the indirect impacts of variables in the HBM. Graffigna et al. (2020) indicated that health engagement on the willingness to get vaccinated was partially mediated by the general attitude toward vaccines. Zheng H. et al. (2021) examined vaccination intention in the United States and found that vaccine-related knowledge had indirect and positive impact on vaccination intention through perceived susceptibility, while doctor-patient communication moderates the negative effect of vaccine knowledge on perceive susceptibility and severity. Despite extensive studies on the indirect impacts of variables in the HBM, researchers argue that variable ordering is currently undefined in the HBM (Jones et al., 2015).

In response to this call, in the current study, we examine the mediating effects of the perceived severity, susceptibility to COVID-19, benefits, and barriers to COVID-19 vaccination on the relationship between government communication and intention to get vaccinated. Government communication is an external cue to an action that can enhance the knowledge and beliefs of individuals about vaccination, especially when considering the high level of credibility and authority the government communication in the context of Chinese society showed during COVID-19. The association between government communication and vaccination intention depends on the perceived severity, susceptibility, benefits, and barriers of individuals. That is, when individuals are exposed to government communication, it will positively motivate them to take the necessary actions to receive the vaccine injection, depending on the level at which they perceive severity, susceptibility of the pandemic, benefits, and barriers to vaccination. Based on the above discussion, we propose the following sub-hypotheses:

H4a: The positive relationship between government communication and vaccination intention is mediated by the perceived severity of COVID-19.
H4b: The positive relationship between government communication and vaccination intention is mediated by the perceived susceptibility to COVID-19.
4Hc: The positive relationship between government communication and vaccination intention is mediated by the perceived benefits of COVID-19 vaccination.
H4d: The positive relationship between government communication and vaccination intention is mediated by the perceived barriers to COVID-19 vaccination.

**FIGURE 1:**
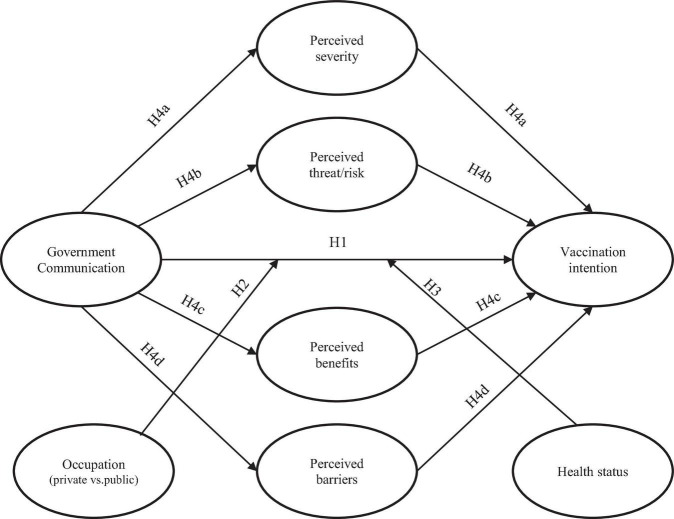
Research framework.

The research framework and hypotheses are summarized and presented in Figure 1.

## Materials and Methods

### Participants and Procedures

This study tested the hypotheses by conducting a cross-sectional online survey of Chinese adults aged 18–59. The survey was conducted from March 13, 2021 to March 22, 2021 by *Sojump*, a top Chinese professional online survey provider with a sampling service of 2.6 million registered users. At the first stage of COVID-19 vaccination campaign in China in March, nine groups of residents with high possibility of occupation exposure aged 18–59 were prioritized to get vaccinated (Zheng W. et al., 2021). The COVID-19 was under control in China in March, with about 10 newly confirmed daily cases reported nationwide from March 6 (one week before the survey) to March 22 (the end of the survey) (Dezan Shira and Associates, 2021). A total of 732 survey invitations to answer questionnaires were randomly sent out, and 602 questionnaires were returned with a response rate of 82.2%. We excluded 45 vaccinated respondents, so the final sample size was 557 (602–45 = 557). Upon completion, the respondents received a gift of approximately one dollar as an incentive. Participants were informed that all information would be kept confidential and that they could withdraw from the study at any time. It took approximately 7 min to complete the questionnaire. Multiple participation was avoided by recording the IP address of the device of each user.

All the items measuring the research variables were slightly revised based on previous studies, after we referred to the operationalization in China to suit the current context of COVID-19. Back translation was performed at this stage, and the translated document was compared with the original English version. An agreement between the back translation and the original source version was achieved. To confirm the reliability of the scales and the proper wording in the questionnaire, a pilot test was conducted among 20 graduate students from a research university in northern China. This study was approved by the Social Science Ethics Committee of a research university in Beijing, China (Approval: UCASS202101). Respondents were informed that their participation was voluntary, and consent was granted prior to completion of the questionnaire. Ethical approval persisted throughout the research. We conducted the research in accordance with the ethical principles, which ensured that the information was collected for research purposes only, was kept confidential, and anonymity of the respondents was guaranteed.

### Measurement

#### Vaccination Intention

COVID-19 vaccine intention was the dependent variable in the study, and it was measured using a four-item scale adapted from previous studies with some changes (Freeman et al., 2020; Sherman et al., 2020). The items included: (1) “I would take a COVID-19 vaccine as a preventative measure as soon as possible”; (2) “I would take a COVID-19 vaccine even if it has not been tested for a long period of time”; (3) “I would get the COVID-19 vaccine regardless of others’ attitudes; and (4) “I would get the COVID-19 vaccine according to the vaccination program.” Participants indicated their intentions using a five-point Likert scale (from not at all = 1 to extremely likely = 5) (Cronbach’s alpha = 0.875).

#### Government Communication

Government communication was measured by the question “To what extent do you think your local government uses following channels to raise public awareness of and recommend protective behavior against the COVID 19?” using a 5-point Likert scale (from not at all frequently = 1 to very frequently = 5). Four channels based on Duan et al. (2020) were used to measure government communication: (1) banners, (2) broadcast, (3) brochure, and (4) WeChat or text messages.

#### Health Beliefs Surrounding COVID-19 Vaccination

Four constructs of HBM included the perceived severity of COVID-19 infection, perceived susceptibility to COVID-19 infection, perceived benefits of a COVID-19 vaccine, and perceived barriers to getting a COVID-19 vaccination. They were measured through five-point scales (from strongly oppose = 1 to strongly agree = 5) to indicate the beliefs of participants about COVID-19 and vaccination, which were adapted from previous studies (Freeman et al., 2020; Wong et al., 2020).

The perceived severity of COVID-19 infection was measured by four items: (1) “COVID-19 is fatal if a person becomes infected”; (2) “A lot of serious complications can be caused by COVID-19”; (3) “Complications can still happen after recovering from COVID-19”; and (4) “I will be very sick if I get COVID-19” (Cronbach’s alpha = 0.877). The Perceived susceptibility to COVID-19 infection was measured by three items: (1) “I am worried about the likelihood of getting COVID-19”; (2) “My likelihood of getting COVID-19 in the next few months is high”; and (3) “Getting COVID-19 is currently a possibility for everyone” (Cronbach’s alpha = 0.874). The perceived benefits of a COVID-19 vaccine were measured by four items: (1) “Taking the COVID-19 vaccine would reduce the risk of COVID-19 and its complications”; (2) “Taking the COVID-19 vaccine is a responsible behavior for the family”; (3) “Getting the vaccine is a sign of contributing to the country and society in fighting against COVID-19”; and (4) “Taking the COVID-19 vaccine will give me complete freedom to get on with life just as before (Cronbach’s alpha = 0.866). The Perceived barriers to vaccination were measured by five items: (1) “I lack basic knowledge about the COVID-19 vaccine”; (2) “I worry about the quality of the COVID-19 vaccine”; (3) “The COVID-19 vaccine has not been sufficiently developed and vaccination is risky”; (4) “I worry about the side effects of the COVID-19 vaccine”; and (5) “Taking the new COVID-19 vaccine will make me feel like a guinea pig” (Cronbach’s alpha = 0.910).

#### Demographic Characteristics

Information was collected on demographics of respondents. Personal information regarding occupation and health status was obtained for multi-group comparisons. Individuals working in government agencies, schools (including students), hospitals, or state-owned enterprises were considered to be from the public sectors (“insiders”), otherwise, those individuals from private, foreign enterprises, or self-employed were grouped as belonging to the private sectors (“outsiders”). The participants were asked to report their current height and weight to assess their objective health statuses *via* body mass index (BMI), which was computed using the standard formula (weight in kilograms/height in meters squared). Following the standard practices (Li S. et al., 2018), individuals with a BMI between 18.5 and 25 are normal, and they have a good objective health status. Individuals with BMI > 25 are overweight or obese, while those with BMI < 18.5 are underweight; therefore, they have a poor objective health status. Participants were also asked to self-assess how “healthy” they were on a five-point Likert scale ranging from 1 (very unhealthy) to 5 (very healthy). Individuals who rated their health as three or below were assigned a poor subjective health status, otherwise, they had a good subjective health status. Other demographic characteristics (gender, education, and age) were used as control variables in the multi-group comparisons. More than half (55.1%) of the 557 participants were female participants. A large portion of the participants has a bachelor’s degree (68.2%), followed by junior college degree (12.0%), master or higher degree (7.9%), junior high school (5.9%), senior high school (5.6%), and elementary school or below (0.4%). Table 1 presents the detailed demographic distribution.

**TABLE 1:** Descriptive statistics of demographic variables.

Characteristics	Frequency	Percent (%)
***Gender***		
Male	250	44.9
Female	307	55.1
***Age***		
18–24	92	16.5
25–30	121	21.7
31–35	121	21.7
36–40	93	16.7
41–45	55	9.9
46–50	29	5.2
51–55	32	5.7
56–60	14	2.5
***Education level***		
Primary school or below	33	5.9
Junior high school	31	5.6
Senior high school	67	12
Junior college	380	68.2
Undergraduate degree	44	7.9
Masters or higher	92	16.5
***Occupation***		
Government	6	1.1
State-owned enterprises	78	14.0
Public institutions (e.g., schools and hospitals)	71	12.8
Students	116	20.8
Self-employed	275	49.4
Others	11	2.0

### Data Analysis

The method, partial least squares structural equation modeling (PLS-SEM), was employed through SmartPLS 3.0 to estimate the simultaneous relationships among multiple constructs in this study. As some constructs in the current study were skewed (e.g., right-skewed usage of government communication), and the model included both the reflective (perceived severity, perceived susceptibility, perceived benefits, perceived barriers, and vaccination intention) and the formative constructs (government communication), PLS, a variance-based SEM, is preferred over a traditional covariance-based SEM for the current analysis (Hair et al., 2017). The significance of the path coefficients was calculated through bootstrapping with 5,000 sub-samples and a two-sided *p*-value less than.1 was considered statistically significant.

As the questions in each questionnaire were answered by one respondent, the results may be susceptible to a common method bias (CMB) (Podsakoff et al., 2003). We created a common method factor (method construct) in the PLS model, including all the indicators of the six principal constructs in the model (Liang et al., 2007). We then calculated the variances of each indicator that was substantially explained by its corresponding principal constructs and the method construct. The average substantive factor loading was 0.738, whereas the average method factor loading was 0.005, resulting in a ratio (substantive variance to method variance) of approximately 139:1. Additionally, the loadings of the principal constructs were all significant (*p* < 0.01), but most of the loadings for the method were not significant (*p* < 0.01). In summary, the relatively small values of loadings and insignificance of the method variance suggest that CMB was not serious.

## Results

The reliability and validity of the measurement scales were assessed in the measurement model first, and the structural model was estimated for the parameters of the construct coefficients and was tested for significance in the second step.

### Measurement Model

Evaluation of the measurement model involves the assessment of reliability and validity for each reflective scale. Reliability assessment includes evaluating internal consistency as Cronbach’s alpha (Alpha) and composite reliability (CR). Specifically, both alpha and CR values exceeded the threshold of.7 (Table 2), ensuring the acceptable reliability for the measurement scales (Hair et al., 2017).

**TABLE 2:** The convergent validity and reliability of the reflective scales.

Constructs	Indicators	Factor loadings	Alpha	CR	AVE
VI: vaccination intention	VI1	0.863***	0.875	0.914	0.727
	VI2	0.842***			
	VI3	0.859***			
	VI4	0.847***			
PSE: perceived severity	PSE1	0.840***	0.877	0.915	0.730
	PSE2	0.842***			
	PSE3	0.860***			
	PSE4	0.875***			
PSU: perceived susceptibility	PSU1	0.915***	0.874	0.920	0.793
	PSU2	0.832***			
PBE: perceived benefits	PSU3	0.921***	0.866	0.908	0.712
	PBE1	0.856***			
	PBE2	0.870***			
	PBE3	0.806***			
	PBE4	0.843***			
PBA: perceived barriers	PBA1	0.795***	0.910	0.933	0.736
	PBA2	0.878***			
	PBA3	0.896***			
	PBA4	0.852***			
	PBA5	0.867***			

*** p < 0.01.

*Cronbach’s alpha (Alpha); composite reliability (CR); average variance extracted (AVE).*

Validity evaluation involves convergent and discriminant validity. The outer loadings of the indicators and the average variance extracted (AVE) were employed to evaluate the convergent validity of the reflective constructs. All loadings were above the threshold of 0.7, and the AVE of each construct was greater than 0.5, as shown in Table 2. Therefore, sufficient convergent validity was confirmed (Hair et al., 2017).

Discriminant validity captures the extent to which one construct is unique and differs from other constructs. The results demonstrated that the loadings were greater than the cross-loadings on the other constructs, and the square roots of the AVE were greater than the correlations among constructs (see Table 3). Therefore, a good discriminant validity can be attained (Hair et al., 2017).

**TABLE 3:** Correlation matrix of the reflective constructs.

	Convergent validity	Discriminant validity				
	AVE	PSE	PSU	PBE	PBA	VI
PSE	0.730	**0.855**				
PSU	0.793	0.243	**0.844**			
PBE	0.712	0.055	0.123	**0.892**		
PBA	0.736	–0.086	–0.420	–0.006	**0.858**	
VI	0.727	0.249	0.492	0.063	–0.610	**0**.**853**

*Average variance extracted (AVE); perceived severity (PSE); perceived susceptibility (PSU); perceived benefits (PBE); PBA: perceived barriers; vaccination intention (VI). The numbers in bold on the diagonal in the matrix of correlation are the square roots of the AVE.*

In summary, these results indicated the good convergent and discriminant validity of the five reflective constructs in the model.

The criteria to evaluate the reliability and validity of the reflective constructs do not apply to formative constructs, whose indicators may denote the independent causes of the construct and do not necessarily covary. Formative indicators are assumed to be error-free, which further suggests that the concept of the internal consistency reliability may not be appropriate (Hair et al., 2017). Alternatively, the evaluation of a formative construct (government communication) in measurement models involves assessing the indicator collinearity and significance, along with the relevance of indicator weights. The variance of inflation factor (VIF) values of all the indicators was below 5, and all outer weights were significantly different from zero (*p* < 0.05) after utilizing 5,000 bootstrapping subsamples, indicating the absence of indicator collinearity and the significance and relevance of indicator weights (see Table 4).

**TABLE 4:** Assessment of formative constructs.

Construct	Indicator	Weights	Standard deviation	Collinearity (VIF)
Government communication	Use of banners	0.259***	0.120	1.978
	Use of broadcast	0.399***	0.121	1.980
	Use of brochures	0.291**	0.115	1.794
	Use of WeChat	0.288***	0.094	1.429

** p < 0.05, *** p < 0.01.

*Variance of inflation factor (VIF).*

### Structural Model

We evaluated the predictive power (e.g., path coefficients and significance) of the structural model. The path coefficients were standardized through bootstrapping with 5,000 samples to test the significance (Hair et al., 2017).

The results showed that government communication had a significant association with vaccination intention (β = 0.105, *p* < 0.01). Therefore, H1 was supported.

To examine H2 and H3, we performed multi-group comparisons of the impacts of government communication on vaccination intention. Researchers have proposed several approaches for multi-group analysis in partial least squares structural equation model (PLS-SEM), and the permutation test is preferred due to its statistical properties in comparing differences of parameters across two groups (Hair et al., 2017). Therefore, the permutation test was employed in this study after demographic variables (gender, education, and age) were controlled. The results showed that the association between government communication and vaccination intention was stronger for individuals from the private sectors than for those from the public sectors (*p* < 0.01) (see Table 5). Therefore, H2 was not supported.

**TABLE 5:** Multi-group comparison test results.

Variable	Groups	Sample size	Coefficient	Difference
Occupation	Public sectors	280	0.028	–0.100**
	Private sectors	277	0.128	
Subjective health status	Good	362	0.088	0.037
	Poor	195	0.051	
Objective health status	Good (BMI = 18.5–25)	397	0.116	0.091*
	Poor (BMI > 25 or BMI < 18.5)	160	0.025	

* p < 0.10, ** p < 0.05.

The results indicated that the association between government communication and vaccination intention was stronger for individuals with objective good health status than poor health status (*p* < 0.10). However, the association between government communication and vaccination intention of individuals with good or poor subjective health status showed no significant difference (see Table 5). Therefore, H3 was only partially supported.

Finally, we examined the mediation effects of health-related beliefs in the relationship between the government communication and the vaccination intention. The results suggested that the government communication was indirectly associated with vaccination intention through perceived severity [indirect effect = 0.16, 95% confidence interval (CI) = (0.120,0.205), *p* < 0.01] (see Table 6). Therefore, H4a was supported. However, government communication was not indirectly associated with vaccination intention through perceived susceptibility to COVID-19 infection (*p* > 0.1). This indicated that H4b was not supported. The results demonstrated that government communication was indirectly associated with vaccination intention through the perceived benefits of vaccination [indirect effect = 0.79, 95% confidence interval (CI) = (0.050,0.113), *p* < 0.01] and the perceived barriers of vaccination [indirect effect = 0.028, 95% confidence interval (CI) = (0.012,0.048), *p* < 0.01] (Table 6). Therefore, H4c and H4d were both supported.

**TABLE 6:** Significance analysis of the mediation effects.

Hypothesized relationship	Indirect effect	95% confidence interval of the indirect effect	*t* value	significance
H4a: GC- > PSE- > VI	0.160***	[0.120, 0.205]	7.385	0.000
H4b: GC- > PSU- > VI	0.000	[–0.010, 0.011]	0.094	0.925
H4c: GC- > BEN- > VI	0.079***	[0.050, 0.113]	4.933	0.000
H4d: GC- > BAR- > VI	0.028***	[0.012, 0.048]	2.947	0.003

*** p < 0.01.

*Government communication (GC); perceived severity (PSE); perceived susceptibility (PSU); perceived benefits (PBE); PBA: perceived barriers; vaccination intention (VI).*

## Conclusion and Discussion

This study confirms that government communication was both directly and indirectly associated with vaccination intention through the mediation of the perceived severity, benefits, and barriers. Multi-group comparisons indicate that government communication was more strongly associated with COVID-19 vaccination intention for individuals from the private sectors than those from the public sectors (e.g., government officials and civil servants). The correlation between government communication and vaccination intention on individuals with a good objective health status is stronger than that of those with a poor objective health status.

Combining the literature on government communication with discussions on perceptions of individuals based on the HBM model, our results offer several important theoretical and practical implications. First, our results contribute to the literature on government communication by offering empirical evidence from a highly collective and hierarchical society. Our results verify that government communication was associated with a heightened level of vaccination intention. Second, we expand the discussion on the HBM model by examining the indirect association between government communication and COVID-19 vaccination through the perceptions of the severity and the susceptibility to COVID-19, benefits of COVID-19 vaccination, and barriers to COVID-19 vaccination. We find that the only perceived severity, benefits, and barriers mediate the relationship between government communication and vaccination intention, while our results do not support the mediation effect of the perceived susceptibility. Third, it is meaningful to note the moderating role of occupation and health status of individuals. Specifically, regarding vaccination intention, our results reveal that government communication carries more weight for individuals from the private rather than public sectors. In addition, our results verify that respondents with a good health status were more susceptible to government communication than those with a poor health status.

### Government Communication and Vaccination Intention in the Chinese Context

A successful vaccination campaign requires the regulatory agencies to an effective communication to successfully engage the public and to ensure the full cooperation with the recommended vaccination procedures. We cannot regard COVID-19 vaccination intention as homogeneous. Rather, we need to conduct a research in specific local contexts with unique features (Yang et al., 2021). We examined a government communication in the unique context in China with a collective culture and strong community governance. This study establishes the internal beliefs about vaccine as a critical bridge between external cues and vaccination intention.

Government communication is increasingly indispensable in facing the global pandemic challenges such as COVID-19, considering that an untimely information disclosure is a great challenge for the health system of China (Xing and Zhang, 2021). Public officials should collaborate closely with the media to improve communication with the public and disseminate relevant information on COVID-19 vaccines. Governments should release timely vaccine information about vaccination strategies, modalities, and accomplishments to enhance transparent and coherent public communication with public engagement (OECD, 2021).

### The Health Belief Model Model and Government Communication

Our results extend the discussion of the HBM model to incorporate indirect interactions among the HBM variables. Our research findings contribute to the current gaps in the literature by specifying the interactions and variable ordering of HBM (Strecher et al., 1997; Skinner et al., 2008), which has important implications for advancing the theory development of a process-oriented HBM model. Our research results verify the parallel mediation model and particularly confirm the mediating effects through the perceived severity, benefits, and barriers, which are consistent with previous research results (Chen et al., 2021). Future research should examine different mediation models of HBM, including parallel mediation (see Skinner et al., 2008), serial mediation (see Nancy et al., 1984), and moderated mediation (see Skinner et al., 2008) in the context of vaccination.

Our results do not support the mediating effect of a perceived susceptibility. This may be due to the current context of study, which is a highly authoritative and hierarchical society in which individuals have a relatively high level of trust in the government. Future studies could examine the possible impact of context cues that influence the perceived susceptibility of individuals, and further verify the indirect impact of this construct. In addition, our results suggest that government communication, as a cue to action, serves as a driver of vaccination intention. It goes beyond most of the existing studies that emphasize the four major factors (i.e., perceived severity, susceptibility, benefits, and barriers), and incorporates the factor of cues to action in developing the extended health belief model (EHBM) (Bylund et al., 2009) with empirical support. Future studies could further examine other external factors of cues to action (e.g., exposure to different types of media and information sources).

### Government Communication and Vaccination Intention Among Different Social Groups

Our results show that government communication is more strongly associated with vaccination intention for individuals from the private sectors than for those from the public sectors. Our results showed that people from the private sectors reported higher levels of vaccination intention (*M* = 3.85, *SD* = 0.67) compared with those from public sectors (*M* = 3.73, *SD* = 0.69), and the difference was statistically significant [*t* (555) = 2.10, *p* < 0.05]. It is possible that the public sector employees are often provided with high-quality medical and health insurance, as well as other social welfare, which made them less aware of the potential severity of the disease. This study indicates that government communication is more strongly associated with the vaccination intention for individuals with an objectively good health status (normal BMI) than those with an objectively poor status. However, this difference does not hold for individuals with different subjective health status. This may suggest that those with a good self-reported subjective health status may unconsciously neglect the vaccination. Our results bridge the gap in the current literature, as most of the previous HBM-based vaccination models focused only on perception of individuals without fully extending the roles of external cues such as government communication (Nancy et al., 1984).

This research provides an illustrative and practical instance of how government communication can be further expanded to study the vaccination intention among various groups. Evidence-based government communication should be implemented to allow vaccines to effectively use for mass immunization, with special attention to the unique groups who may have special needs. For example, the employed are more likely to get COVID-19 vaccination than the unemployed in US (Malik et al., 2020).

This study shows that both personal and environmental factors can facilitate an in-depth understanding of vaccination intention. The results provide preliminary evidence of a nuanced relationship between government communication and vaccination intention, which, in its present state, is a nascent yet emerging field that bears potential for the future research.

### Limitations and Implications for Future Research

This study has several limitations. First, this study relies on a cross-sectional design. A longitudinal design is recommended for future studies to test the causal effects between variables of government communication and vaccination intention. Second, our survey was conducted in China, a unique cultural society in Asia. Accordingly, researchers should be cautious in applying our findings to Western cultures. We recommend for future studies to examine the relationship between vaccination intention and government communication in other cultural and institutional contexts, acknowledging the possible impact of the contextual cues on the intention and actions of individuals.

The coronavirus disease (COVID-19) and the rapid development of its vaccine have, thus, sparked far more interest than that of any other global pandemic, such as influenza and measles, in the human history. This study provides an important validation of the HBM model as a parsimonious and a powerful model for examining the vaccination intention and related behavior. We recommend future research to further examine different types of global pandemics and vaccinations, considering the emerging landscape of media both at the local and global levels.

## Data Availability Statement

The raw data supporting the conclusions of this article will be made available by the authors, without undue reservation.

## Ethics Statement

The studies involving human participants were reviewed and approved by the Social Science Ethics Committee at a Research University, Beijing, China (Approval: UCASS202101). Respondents had been informed that their participation was voluntary, and consent was granted on the completion of the questionnaire.

## Author Contributions

LS: manuscript writing, data analysis, and manuscript revision. ZD: conceptualization and survey execution. JD: literature review and manuscript revision. All authors contributed to the article and approved the submitted version.

## Conflict of Interest

The authors declare that the research was conducted in the absence of any commercial or financial relationships that could be construed as a potential conflict of interest.

## Publisher’s Note

All claims expressed in this article are solely those of the authors and do not necessarily represent those of their affiliated organizations, or those of the publisher, the editors and the reviewers. Any product that may be evaluated in this article, or claim that may be made by its manufacturer, is not guaranteed or endorsed by the publisher.
